# Identification of cell wall binding domains and repeats in *Streptococcus pneumoniae* phage endolysins: A molecular and diversity analysis

**DOI:** 10.1016/j.bbrep.2024.101844

**Published:** 2024-10-14

**Authors:** Tahsin Khan, Shakhinur Islam Mondal, Araf Mahmud, Daniyal Karim, Lorraine A. Draper, Colin Hill, Abul Kalam Azad, Arzuba Akter

**Affiliations:** a Department of Genetic Engineering and Biotechnology, Shahjalal University of Science and Technology, Sylhet, Bangladesh; b APC Microbiome Ireland, University College Cork, Cork, Ireland; c School of Microbiology, University College Cork, Cork, Ireland; d Department of Biochemistry and Molecular Biology, Shahjalal University of Science and Technology, Sylhet, Bangladesh

**Keywords:** Endolysin, Alternatives to antibiotics, *Streptococcus pneumoniae*, Drug resistance

## Abstract

*Streptococcus pneumoniae* (pneumococcus) is a multidrug-resistant pathogen associated with pneumonia, otitis media, meningitis and other severe complications that are currently a global threat to human health. The World Health Organization listed *Pneumococcus* as the fourth of twelve globally prioritized pathogens. Identifying alternatives to antibiotic therapies is urgently needed to combat *Pneumococcus*. Bacteriophage-derived endolysins can be used as alternative therapeutics due to their bacterial cell wall hydrolyzing capability. In this study, *S. pneumoniae* phage genomes were screened to create a database of endolysins for molecular modelling and diversity analysis of these lytic proteins. A total of 89 lytic proteins were curated from 81 phage genomes and categorized into eight groups corresponding to their different enzymatically active (EAD) domains and cell wall binding (CBDs) domains. We then constructed three-dimensional structures that provided insights into these endolysins. Group I, II, III, V, and VI endolysins showed conserved catalytic and ion-binding residues similar to existing endolysins available in the Protein Data Bank. While performing structural and sequence analysis with template lysin, an additional cell wall binding repeat was observed in Group II lysin, which was not previously known. Molecular docking performed with choline confirmed the existence of this additional repeat. Group III endolysins showed 99.16 % similarity to LysME-EF1, a lysin derived from *Enterococcus faecalis*. Furthermore, the comparative computational analysis revealed the existence of CBDs in Group III lysin. This study provides the first insight into the molecular and diversity analysis of *S. pneumoniae* phage endolysins that could be valuable for developing novel lysin-based therapeutics.

## Highlights

• Explored *S. pneumoniae* phage genomes to create an endolysin database.
• Identified eight distinct endolysin groups, each with unique domain architecture.
• Found an additional cell wall binding repeat in Group II lysin by molecular analysis.
• Identified CBD in Group III lysin through comparative computational analysis.
• Offers valuable insights for novel lysin-based therapeutics.

## 1 Introduction

The discovery of antibiotics is a landmark in the history of medicine, which revolutionized the battle against bacterial infection and saving countless lives. However, the “golden era” of antibiotic discovery, spanning from the 1930s–1960s, came to an end as antibiotic-resistant pathogens emerged at a faster rate than new antibiotics could be developed [1]. This shift has raised concerns about losing the battle against bacterial infections [2,3]. Factors such as clinical misuse, ease of access, poor quality control, lack of surveillance, overuse in animal farming, inadequate sanitation, and a decline in antibiotic discovery research have accelerated the emergence of multidrug-resistant (MDR) bacterial strains [4]. The global threat of “superbugs” and “super resistance”, characterized by multiple gene mutations, has resulted in higher morbidity and mortality rates [5].

There are several mechanisms of bacterial antibiotic resistance, including single nucleotide changes, structural modifications, horizontal gene transfer, antibiotic inactivation, efflux pumps, target site alteration and metabolic bypass [6]. Pathogens like *Staphylococcus aureus*, *Acinetobacter baumannii*, *Mycobacterium tuberculosis* and *Streptococcus pneumoniae* are recognized globally for their MDR capabilities [[7], [8], [9], [10]], among many more [5]. Without intervention, it is estimated that antibiotic-resistant infections could lead to 10 million deaths per year and a $100.2 trillion loss in global GDP by 2050 [11].

*S. pneumoniae* (*Pneumococcus*) is a Gram-positive, extracellular, opportunistic pathogen colonizing the upper respiratory tract. It causes disease such as pneumonia, meningitis, bacteremia, and otitis media, especially in infants, the elderly and immunocompromised individuals [12]. The incidence is highest in individuals under two and adults over 60 years of old [13,14]. Nasopharyngeal colonization facilitates horizontal spread, contributing to community-wide transmission [15]. The pathogen can invade the lower respiratory tract or bloodstream, leading to severe inflammatory diseases [16], with variations in acquisition and carriage based on age, geography, genetics, and socioeconomic circumstances [17]. Pneumonia remains a leading cause of death in children under five, accounting for 1.6 million deaths each year, and pneumococcal diseases remain the top vaccine-preventable cause of death globally [18]. Despite advances in vaccines and antibiotics, the emergence of antibiotic-resistant *S. pneumoniae* is a significant global concern due to the organism’s ability to acquire exogenous DNA and remodel its genome [12].

In 2017, the World Health Organization (WHO) included *S. pneumoniae* in a list of priority pathogens due to its increasing antibiotic resistance. The first reported case of penicillin-resistant *S. pneumoniae* was in 1967 in Australia, followed by South Africa in 1977 [19,20]. By the late 1970s, multidrug-resistant pneumococcal strains were reported globally, and the Centers for Disease Control and Prevention (CDC) now estimates that 30 % of pneumococcal infections are resistant to at least one antibiotic. Resistant strains are responsible for an estimated 1.2 million illnesses annually in the USA alone. Mechanisms of resistance in *S. pneumoniae* have been well documented [21], involving beta-lactams, macrolides, lincosamides, fluoroquinolones, tetracyclines, and trimethoprim resistance due to various genetic adaptations [22].

The limitations of conventional antibiotic discovery have led to increased exploration of alternative novel approaches to combat antibiotic-resistant bacteria [23,24]. Czaplewsk and colleagues highlighted bacteriophage-based therapies as a top priority among 19 alternatives to antibiotics [25]. Bacteriophage or phages, are viruses that infect bacteria and exhibit two life cycles: lysogenic and lytic. While lysogenic phages integrate their genetic material into the bacterial genome, lytic phages result in the destruction of the bacterial host [26]. Phage-derived endolysins, are hydrolytic enzymes produced during the lytic cycle, are particularly promising as they degrade the bacterial cell wall, causing rapid bacterial lysis and death [27,28]. Endolysins are highly specific to their target bacteria, preserving beneficial microbiota and demonstrating low resistance potential [[29], [30], [31]]. Moreover, endolysins can act synergistically with existing antibiotics, enhancing their efficacy [32].

Endolysins, particularly those targeting Gram-positive bacteria like *S. pneumoniae*, typically consist of an enzymatically active domain (EAD) and a cell wall-binding domain (CBD) [33]. Based on their enzymatic activity, endolysins can be classified into several types depending on the chemical bonds they cleave, including amidases, lysozymes, and endopeptidases [34]. Some EADs, such as CHAP (cysteine, histidine-dependent amidohydrolases/peptidases) domains, exhibit dual functionality, acting as both amidases and endopeptidases [35]. Several *S. pneumoniae* phage endolysins, such as Cpl-1, Ejl, and Cpl-7, have been well characterized and shown to be effective both *in vitro* and *in vivo* [36]. Recent advancements have explored engineered endolysins with enhanced activity. For example, chimeric enzymes combining domains from different endolysins, such as Cpl-711, have demonstrated superior antibiofilm and antimicrobial effects [37]. Their ability to disintegrate biofilms and synergistic effects with antibiotics further enhance their therapeutic potential [38,39]. Moreover, clinical trials on phage-derived lysins have shown promising outcomes, further supporting the potential of endolysins as a novel therapeutic class [40].

In this study, we explored *S. pneumoniae* bacteriophage sequences to identify potential endolysins. Through computational analyses at the proteomic level, we examined the diversity and molecular characteristics of these endolysins. Structural insights were accomplished through the sequence and structural alignment to identify new repeats and domains that could contribute to future therapeutic applications.

## 2 Methods

### 2.1 Database creation

*Streptococcus pneumoniae* specific phage genomes were retrieved from Uniprot (query: *Streptococcus* phage and *Streptococcus* virus) and NCBI Genome database (query: Virus). All complete and partial phage genomes were screened for lytic proteins using the following keywords: “lysin”, “lysozyme”, “murein”, “amidase”, “cell wall hydrolase,” “peptidase,” and “peptidoglycan”. Metadata associated with selected *S. pneumoniae* phages, such as genome accession number, genome size, topology and completeness, phage family, source of isolation, continent and bacterial host strain were retrieved from UniProt, NCBI and ENA archive databases. Corresponding lytic proteins with accession number and amino acid length were also added to the database.

### 2.2 Phylogenetic tree of lytic proteins

The phylogenetic analysis of lytic proteins was performed using the IQ-TREE web server (http://iqtree.cibiv.univie.ac.at), a tool that constructs maximum likelihood phylogenetic trees. The maximum likelihood phylogenetic tree was built using IQ-TREE 2 multicore version 2.0-rcl v2.0 with the posterior mean site frequency (PMSF) [41]. The resulting phylogenetic tree was visualized through the Interactive Tree of Life (iTOL) v6 server (https://itol.embl.de), which provides a user-friendly platform for visualizing large phylogenetic trees and allows for the customization of various visual elements [42]. Additionally, a sequence similarity network (SSN) was generated for the lytic proteins using the Enzyme Function Initiative–Enzyme Similarity Tool (EFI–EST). SSNs help visualize clustering patterns based on sequence similarity, and the network was visualized in Cytoscape v3.7.1, which is a widely used software for molecular interaction network visualization [43].

### 2.3 Functional domain prediction

To predict the functional domains present in the lytic proteins, their amino acid sequences were submitted to the Pfam Database (http://pfam.xfam.org) [44]. Pfam is a large collection of protein families represented by multiple sequence alignments and hidden Markov models (HMMs). This tool is critical for predicting the conserved domains of proteins, which are important for understanding their functional properties. The results from Pfam were used to categorize lytic proteins into different groups based on phylogenetic relationships and the EADs they contained.

### 2.4 Homology modeling and structural optimization through Molecular Dynamics simulation of lytic proteins

Swiss-Model server (https://swissmodel.expasy.org) [45] was used to design the Three Dimensional (3D) structural models of lytic proteins. Consensus template proteins available in Protein Data Bank (RSCB PDB) (https://www.rcsb.org) were selected based on the blast results generated by the Swiss-Model server considering the relevant functions. Proteins that could not be modeled using the Swiss-Model server were instead modeled by AlphaFold [46]. The energy minimization of the 3D structural models was conducted through Molecular Dynamics simulation (MDS) in GROMACS with the aid of the AMBER14 force field [47]. The system was initially cleaned and optimized. The TIP3P model was applied where Na/Cl ions were added with a density of 1.012 gm/cm^3^. The Particle Mesh Ewald was used to calculate long-range electrostatic interaction with a distance of 8 Å. The physiological system of the complex was 298 K with a pH of 7.0 and 0.9 % NaCl. A cubic simulation cell was created (126.4688 Å × 126.4688 Å × 126.4688 Å), and periodic boundary conditions were maintained. Then MDS was run with a time step of 2.50 fs. Finally, root mean square deviation (RMSD), and root mean square fluctuation (RMSF) were analyzed to check the quality of constructed 3D structures.

### 2.5 Molecular docking of group II lysin and autolysin LytA protein

The choline molecule (PubChem CID 305) and crystal structure of LytA (PDB 4X36) of *S. pneumoniae* were retrieved and prepared in PyMol softwere for molecular docking. A rigid docking was conducted in PyRx software to check the binding affinity of choline with CBDs of Group II lysin and LytA protein. The gridbox (1.57∗1.89∗2.48 Å) was kept to confine the last two CBD repeats of LytA and Group II lysin. The docked complexes were visualized in Chimera v1.14 software [48] and Discovery Studio 2019 software.

## 3 Results

### 3.1 *Streptococcus pneumoniae* phages and their lytic proteins

A database of 103 lytic proteins from 94 phage genomes (85 complete and 9 partial) was initially obtained from the UniProt and NCBI databases. Fourteen lytic proteins were excluded from the study due to partial sequences or incomplete EAD. Hence, the final database comprised 89 lytic proteins from 81 phage genomes. Among these, *Siphoviridae* was the most predominant family, representing 75 genomes, while *Salasmaviridae* (2/81) and *Myoviridae* (1/81), and three unclassified phages were also identified. All genomes were double-stranded DNA, with the majority being linear genomes (78/81). Geographically, 34 phage genomes were isolated from Europe, 21 from North America, 18 from Asia, 9 from Africa, 2 from South America and 1 from an unknown origin. Most genomes were isolated from clinical settings, with the exception of three that had unknown isolation sources. Details of the database are available in Supplementary Table 1.

### 3.2 Phylogenetic and sequence similarity analysis of lytic proteins

A phylogenetic tree of 89 lytic proteins was constructed using IQ-TREE and generated eight distinct Groups (I-VIII). The result was consistent when the lysin sequences were categorized based on EADs and CBDs (Fig. 1). The lysins clustered by sequence similarity into five distinct clusters and three singletons (Fig. 1).

**Fig. 1:**
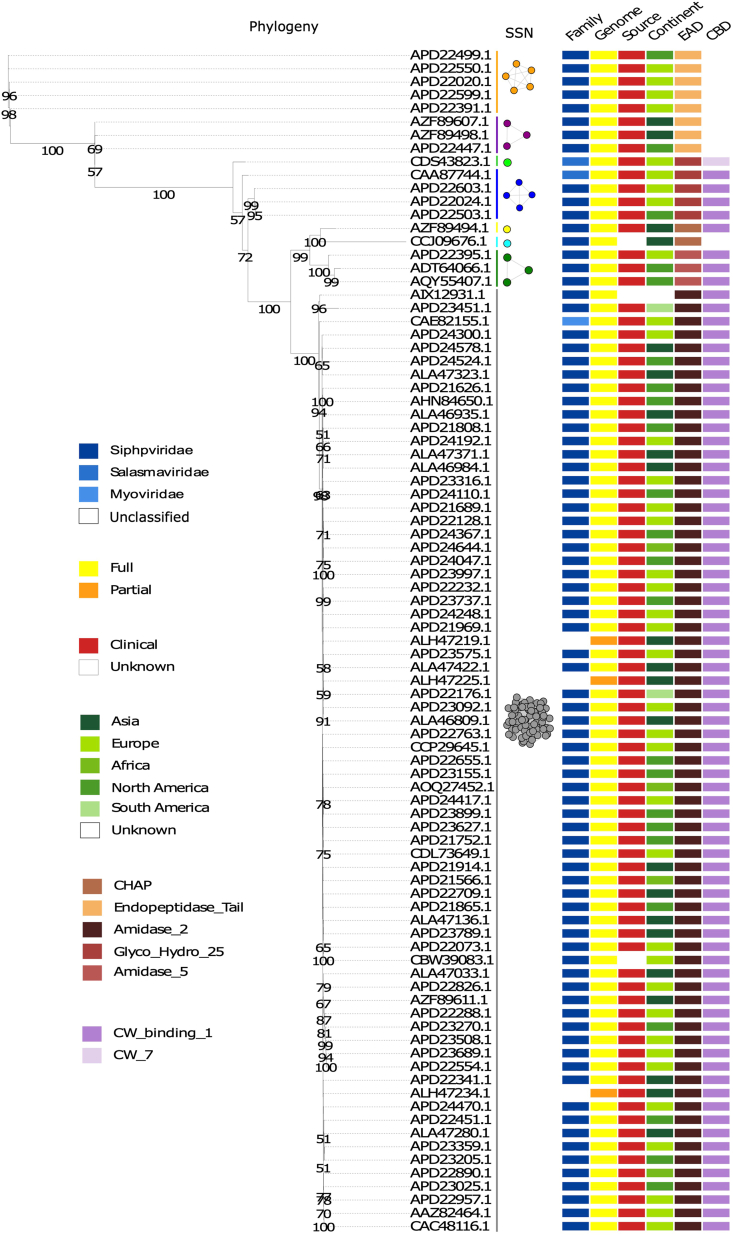
Evolutionary and sequence similarity network analysis of *S. pneumoniae* phage lysins. In both analyses, eight groups were clustered. Phylogenetic tree built for lysin protein sequences from *S.* p*neumoniae* phages, and identification of lysin clusters according to the similarities of lysin sequences. Group colour codes, Gray: Group I; Yellow: Group II; Cyan: Group III; Green: Group IV; Blue: Group V; Lime: Group VI; Purple: Group VII; and Orange: Group VIII. (For interpretation of the references to colour in this figure legend, the reader is referred to the Web version of this article.)

Group I contained 71 lytic proteins, with 67 of these proteins being 318 amino acids (aa) in length, while the remaining proteins varied between 204 and 323 aa. All proteins in Group I harbor an N-terminal catalytic Amidase_2 domain (PF01510) and six C-terminal CW_binding_1 repeats (PF01473) (Fig. 2), except for the shortest protein (APD24470.1), which only contains the catalytic domain. Group II consisted of a single lytic protein containing 288 aa having an N-terminal CHAP (cysteine, histidine-dependent amidohydrolases/peptidases) domain and five C-terminal cell wall binding repeats (CW_binding_1 domains). Similarly, Group III also had a single lytic protein, 237 aa in length, with an N-terminal CHAP domain but no CBD. Group IV included three lytic proteins, each between 295 and 296 aa, featuring an N-terminal amidase_5 domain (PF05382) and six C-terminal cell wall binding repeats (CW_binding_1 domain) (Fig. 2). Group V contained four proteins, ranging from 333 to 339 aa, all of which have an N-terminal Glycosyl hydrolase family 25 (Glyco_hydro_25) domain (PF01183) and six C-terminal cell wall binding repeats (CW_binding_1 domain). Group VI comprised a single 342 aa long lytic protein, with the same EAD found in Group V, but with three C-terminal CW_7 (PF08230) repeats as its CBD. Groups VII and VIII each contained proteins with Prophage_tail domains (PF06605) as their EAD (Fig. 2). The main distinction between these groups was their length: Group VII lytic proteins ranged from 455 to 458 aa, while Group VIII lytic proteins were 450 aa long. There are three and five lytic proteins in Group VII and Group VIII, respectively. The CW_binding_1 repeats within each group showed high sequence similarity (>95 %). To simplify the analysis, a representative lytic protein from each group was selected for further study (Table 1).

**Fig. 2:**
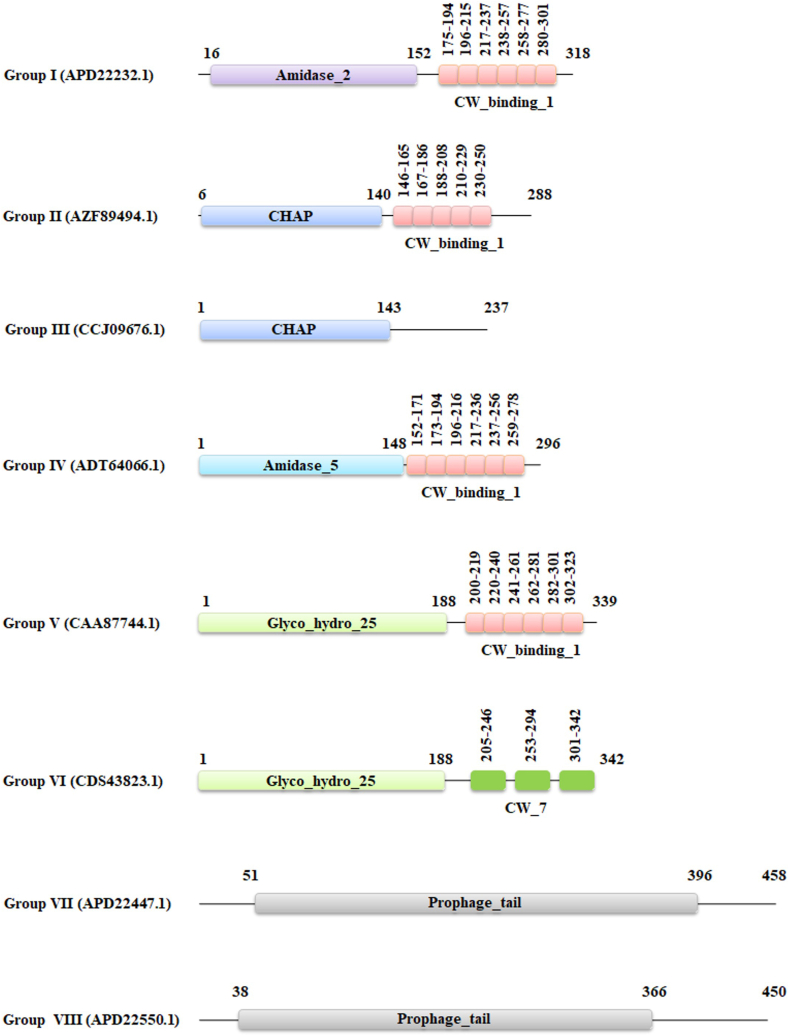
Functional domains present in *S. pneumoniae* phage lysins. The numbers above the rectangles correspond to amino acid residue positions. The accession number of the representative lytic protein of each group is displayed in parentheses.

**Table 1:** Representative *Streptococcus pneumoniae* phage endolysins from eight groups.

Group No.	Genome Accession	Phage	Protein Name	Protein Accession	AA	Family	Topology	Genome Base Pair	Source	Continent	EAD	CBD
I	KY065461	*Streptococcus* phage IPP20	Lytic amidase	APD22232.1	318	Siphoviridae	Linear	37,441	Clinical	Europe	Amidase_2	CW_binding_1
II	MK044828	*Streptococcus* phage 33,888	Lytic amidase	AZF89494.1	288	Siphoviridae	Linear	34,256	Clinical	Asia	CHAP	CW_binding_1
III	HE962497	*Streptococcus* phage SP-QS1	N-acetylmuramoyl- l-alanine amidase	CCJ09676.1	237	Siphoviridae	Circular	58,305	Unknown	Asia	CHAP	
IV	HQ268735	*Streptococcus* phage Dp-1	Endolysin	ADT64066.1	296	Siphoviridae	Linear	56,506	Clinical	North America	Amidase_5	CW_binding_1
V	Z47794	*Streptococcus* phage Cp-1	Lysozyme	CAA87744.1	339	Salasmaviridae	Linear	19,343	Clinical	Europe	Glyco_hydro_25	CW_binding_1
VI	LK392619	*Streptococcus* phage CP-7	Phage endolysin (lytic lysozyme; muramidase)	CDS43823.1	342	Salasmaviridae	Linear	19,741	Clinical	Europe	Glyco_hydro_25	CW_7
VII	KY065465	*Streptococcus* phage IPP24	Putative membrane metallo- endo-peptidase	APD22447.1	458	Siphoviridae	Linear	34,638	Clinical	North America	Prophage_tail	
VIII	KY065467	*Streptococcus* phage IPP26	Endolysin	APD22550.1	450	Siphoviridae	Linear	34,001	Clinical	Europe	Prophage_tail	

### 3.3 Homology modeling, model optimization and structural insights of lytic proteins

#### 3.3.1 Group I lysin

**Fig. 3:**
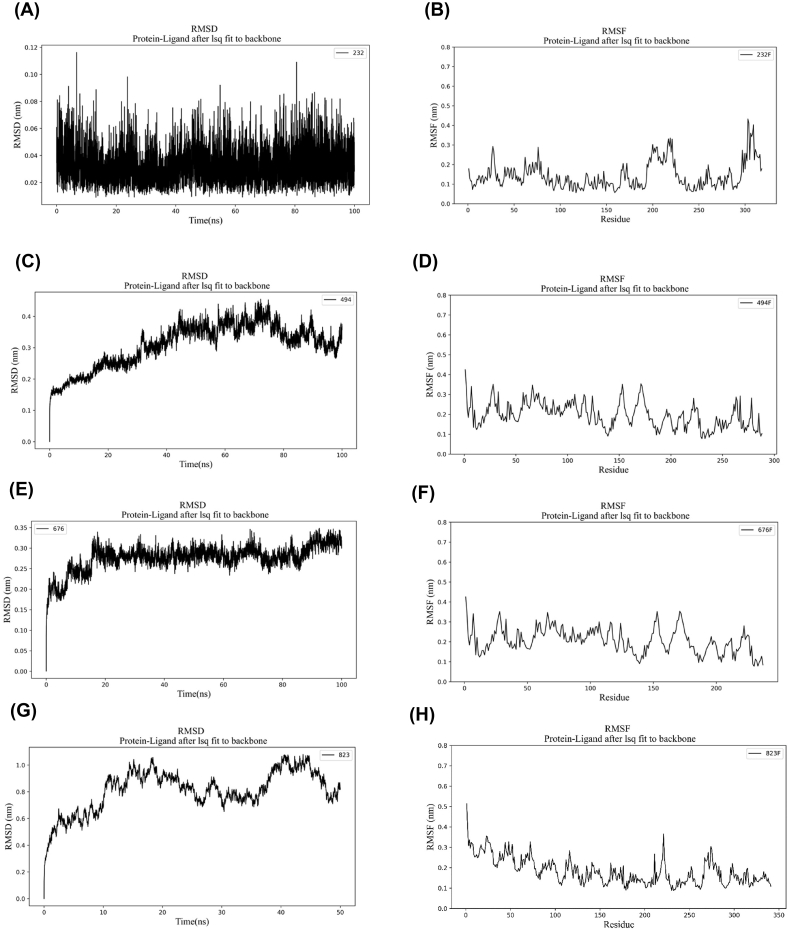
Molecular dynamics simulation to achieve energy-minimized highly stable 3D structures. The RMSD plot for lysins of Groups I (A), II (C), III (E) and VI (G) are shown. The RMSF plot for lysins of Group I (B), II (D), III (F) and VI (H) are shown.

**Fig. 4:**
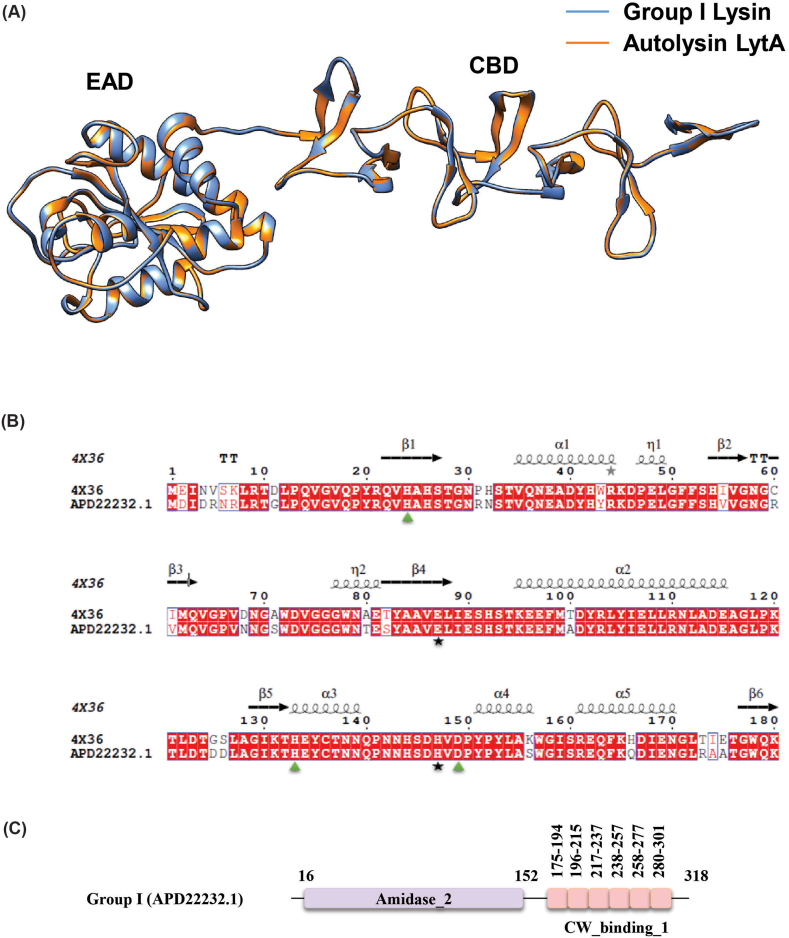
Superimposition and sequence similarity between LytA (PDB 4X36) and Group I lysin. A) Blue and orange colour indicates Group I lysin and LytA lysine, respectively. B) Alignment of Amidase_2 domain of LytA of *S. pneumoniae* and Group I lysin (APD22232.1). Conserved Zn^2+^ binding residues (H26, H133 and D149) are indicated in green triangles whereas catalytic residues (E87 and H147) are indicated in black stars. C) Domain architectural representation of Group I lysins. (For interpretation of the references to colour in this figure legend, the reader is referred to the Web version of this article.)

The lytic protein of Group I showed 89.31 % similarity to the previously characterized crystal structure of the LytA protein (PDB 4X36) (Table 2) from *S. pneumoniae* which contains an N-terminal Amidase_2 domain and six C-terminal choline-binding repeats (CW_binding_1). Using PDB 4X36 as a template, the three-dimensional structure (3D) of the Group I lysin was modeled. Molecular dynamics (MD) simulation, based on RMSD and RMSF analyses over 100 ns, indicated that the Group I lysin was structurally stable after 40 ns (Fig. 3A and B). Structural superimposition of the Group I lysin with the LytA protein revealed that key catalytic residues (E87, H147) in the Amidase_2 domain were identical, while Zn^2+^ binding residues (H26, H133 and D149) were also conserved. Additionally, the six choline-binding repeats in both proteins were found to be structurally similar, suggesting that Group I lysin is likely to exhibit similar choline-binding activity and function to the LytA protein in *S. pneumoniae* (Fig. 4).

**Table 2:** Templates selected to design 3D structure of lytic proteins.

Group	Accession No	Domain		Length (aa)	Full length Template		Template EAD		Template CBD	
		EAD	CBD		PDB ID	Similarity (%)	PDB ID	Similarity (%)	PDB ID	Similarity (%)
I	APD22232.1	Amidase_2	CW_binding_1	318	4X36	89.31				
II	AZF89494.1	CHAP	CW_binding_1	288			5UDM	54.74	4IWT	56.03
III	CCJ09676.1	CHAP		237	6IST	99.16				
VI	CDS43823.1	Glyco_hydro_25	CW_7	342			2IXU	85.05	5I8L	100

aa: Amino acid, EAD: Enzymatically Active Domain, CBD: Cell Wall Binding Domain, PDB: Protein Data Bank.

#### 3.3.2 Group II lysin

**Fig. 5:**
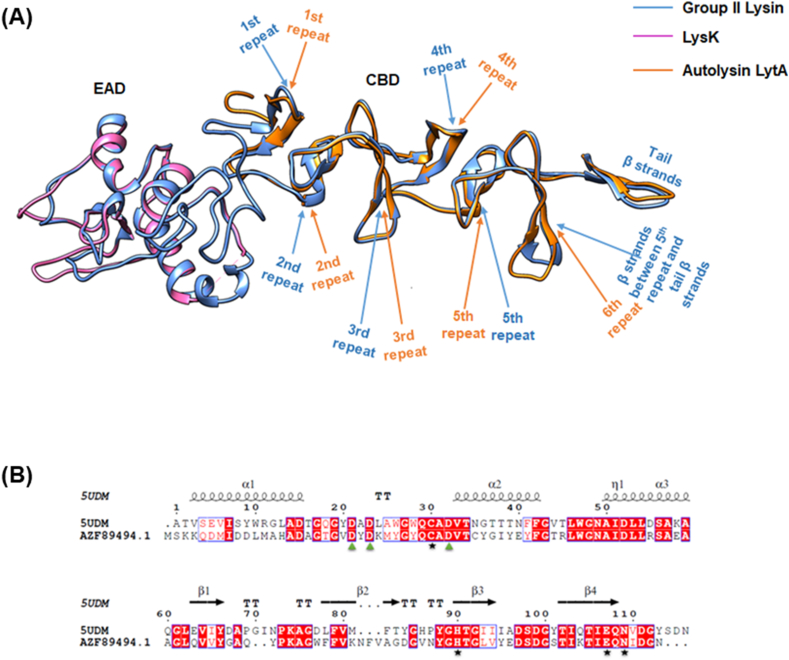
Superimposition of Group II lysin with LysK endolysin (PDB 4CSH) and sequences alignment (EAD) with LysK protein and PlyPy endolysin (PDB 5UDM). A) Blue indicates Group II lysin (whole), pink indicates EAD of LysK and orange indicates CBD of LytA (PDB 4IWT). Position of CBD repeats in Group II lysin and LytA protein are indicated in blue and orange arrows respectively. B) Conserved Ca^2+^ binding residues (D22, D24 and D33) are indicated in green triangles whereas catalytic residues (C31, H92, E109 and N111) are indicated in black stars. (For interpretation of the references to colour in this figure legend, the reader is referred to the Web version of this article.)

Two templates were found for Group II lytic protein. The CHAP domain of Group II lysin showed 51.3 % similarity with endolysin LysK (PDB 4CSH) from *S. aureus* bacteriophage K, and 54.74 % similarity with the phage-associated cell wall hydrolase PlyPy protein (PDB 5UDM) from *S. pyogenes*, while CW_binding_1 repeats showed 56.04 % similarity with PDB 4IWT, the cell wall binding domain of the LytA protein from *S. pneumoniae*. Due to higher similarity, PDB 5UDM was selected for model generation, while PDB 4CSH was used for structural analysis. Using these templates, a 3D structure of the Group II lytic protein was generated. Stability of the Group II lysin was observed at 45 ns and continued until 78 ns (Fig. 3C and D). Structural superimposition of Group II lysin with PlyPy and LysK proteins signifies the conservancy of catalytic residues (C31, H92, E109 and N111) and Ca^2+^ binding residues (D22, D24 and D33) in the CHAP domain. Moreover, the Pfam server predicted five choline-binding repeats (CBDs), similar to LytA, with structural alignment indicating an overlap of the 6th choline-binding repeat of LytA with two β strands of the Group II lysin (Fig. 5). Since these β strands were not predicted to be a choline-binding repeat in Group II lysin protein, molecular docking was performed between CBD of Group II lysin protein and choline to test whether these β strands have the potentiality to bind choline in a similar fashion of LytA CBD (discussed in the discussion section).

**Fig. 6:**
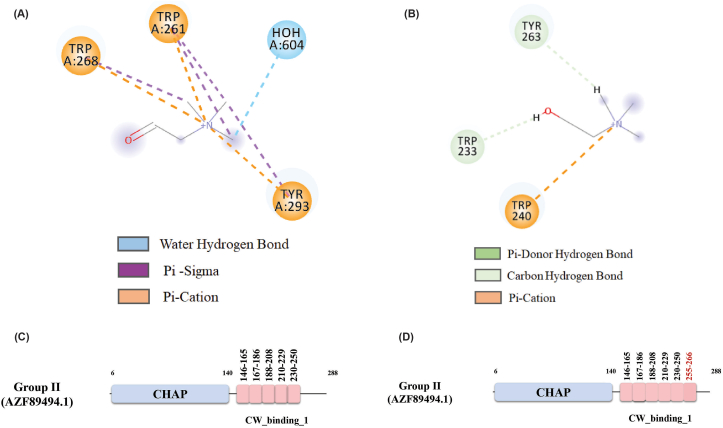
Molecular docking of Choline with CBDs of LytA and Group II lysin. A) Choline docked between 5th and 6th repeat of LytA CBD whereas choline forms bonds with aromatic amino acids between 5th and 6th CW_binding_1 repeat. B) Choline docked with 5th and presumed 6th repeat of Group II lysin CBD whereas choline forms bonds with aromatic amino acids of 5th CW_binding_1 repeat and β strands in between 5th CBD repeat and tail β strands. C) Predicted CBD repeats of Group II Lysin. D) 6th CBD repeat in Group II Lysin predicted by molecular docking approach.

Further analysis revealed that structural superimposition and sequence alignment of the CBDs of LytA and Group II lysin showed two β strands (6th CBD repeat) overlapped (Fig. 5A). Though domain prediction databases did not identify the 6th CBD repeat in Group II lysin, molecular docking results confirmed its presence. A binding affinity of 8.9 Kj/mol and 7.6 Kj/mol was observed for Choline-LytA and Cholin_Group II lysin protein respectively. Aromatic residues (Trp261, Trp268, Tyr293) in 5th (aa 258–277) and 6th (aa 280–301) repeats of CW_Binding_1 domain of LytA protein interact with choline by pi (π) - cation and pi-sigma (π-Σ) bonds (Fig. 6A). Same residues with corresponding position (Trp233, Trp240, Lys263) interacted with 5th (aa 230–250) and presumed 6th (aa 255–266) repeat of Group II lysin but the binding interaction was different such as Trp240 formed pi-cation bond with choline, Trp233 and Tyr263 had π-donor H bonds with choline whereas these were pi - cation and pi-sigma bonds in CBD of LytA protein (Fig. 6B). The bond changes are due to deletion of a couple of residues from the presumed 6th CW_binding_1 repeat of Group II lysin. These findings suggest the presence of an additional CW_binding_1 repeat, the 6th repeat (aa 255–266), in Group II lysin, bringing the total number of CW_binding_1 repeats to six (Fig. 6C and D).

#### 3.3.3 Group III lysin

**Fig. 7:**
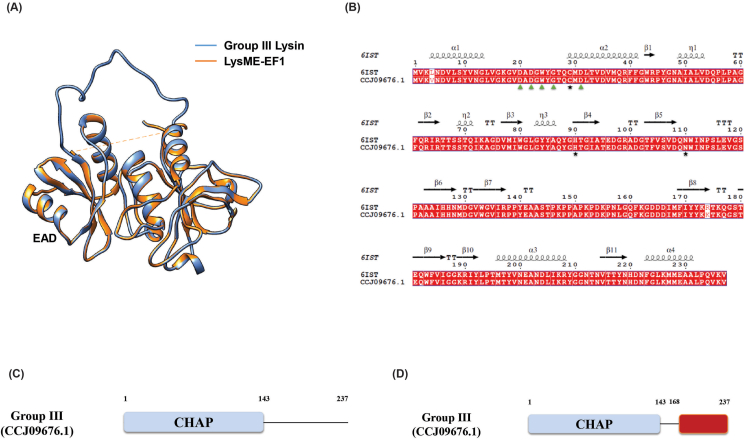
Superimposition and sequence alignment of Group III lysin with *E. faecalis* phage lysin LysME-EF1 (PDB 6IST). A) Blue and orange colour indicates Group III and LysME-EF1 lysin, respectively. B) Conserved Ca^2+^ binding residues (D20, D22, D24, G26 and D31) and catalytic residues (C29, H90 and N110) are indicated in green triangles and black stars, respectively. C) Domain predicted before structural and sequence alignment. D) Domain noticed after structural superimposition, sequence alignment and literature review. (For interpretation of the references to colour in this figure legend, the reader is referred to the Web version of this article.)

Group III lytic protein showed 99.16 % similarity with the full-length LysIME-EF1 protein (PDB 6IST) of *Enterococcus faecalis* phage and, a 3D structure was generated accordingly. The structure remained stable after 18 ns and with a slight increase observed at 90 ns (Fig. 3E and F). Initial analysis revealed that Group III lysin has a catalytic CHAP domain but lacks a CBD. Interestingly, it shares a 99.16 % sequence similarity with *E. faecalis* phage lysin LysIME-EF1. Computational analysis of Group III lysin and LysIME-EF1 confirmed the presence of the CHAP domain with conserved catalytic triad (C29, H90, N110) and Ca^2+^ binding residues (D20, D22, W24, G26, D31). The CHAP domain is located in the same position in both lytic proteins (Fig. 7A). Recent crystallographic analysis of LysIME-EF1 endolysin identified a CBD alongside the EAD [49]. Sequence alignment, apart from one mismatch (Arg174Lys), revealed that the CBD (168–237 aa) is fully conserved between Group III lysin and LysIME-EF1 (Fig. 7B). As a result, the predicted domain architecture of Group III lysin was revised to include the newly identified CBD module (Fig. 7C and D). The newly identified CBD of Group III lysin opens new possibilities for exploring ligand identification in the cell wall of *S. pneumoniae* and further research on this CBD could help design chimeric lysin with broad-spectrum cell wall binding activities.

#### 3.3.4 Group V and VI lysins

**Fig. 8:**
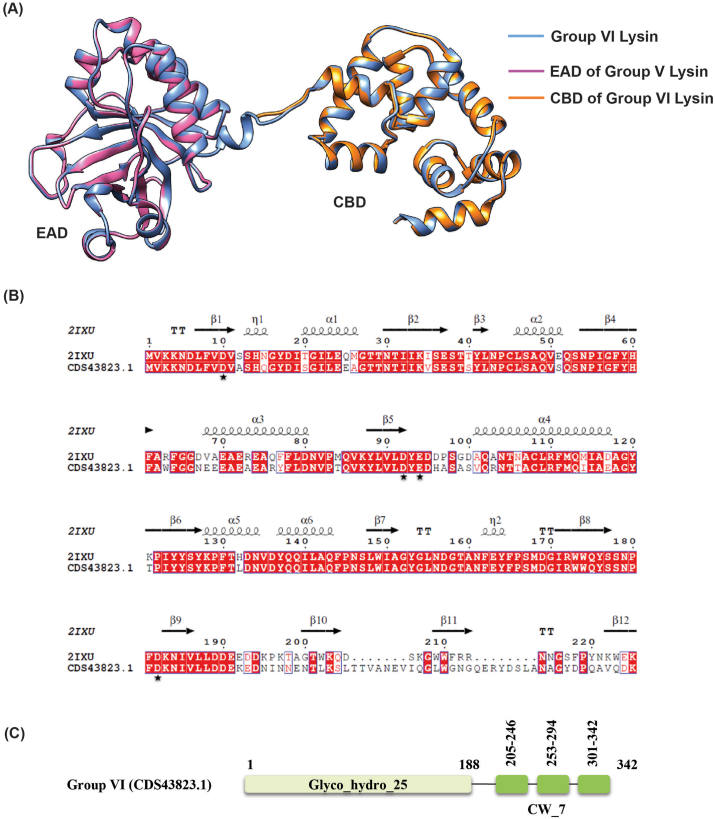
Superimposition and sequence alignment of Group V and VI lysin (EADs). A) Blue indicates Group VI lysin (whole), pink denotes Group V lysin (EAD) and orange indicates the original CBD crystal structure of Group VI lysin. B) Conserved catalytic residues (D10, D92, E94 and D182) are indicated in black stars. C) Domain architectural representation of Group VI lysins. (For interpretation of the references to colour in this figure legend, the reader is referred to the Web version of this article.)

Both Group V and VI lysins contain the Glyco_hydro_25 domain as the enzymatic activity domain (EAD). For Group V, the lytic protein Cpl-1 was previously modeled through X-ray diffraction (PDB 2IXU), while the crystal structure of the CW_7 domain (PDB 5I8L) provided insights into the structure of Group VI lysin. The EAD of Group VI lysin was modeled based on its similarity to Group V lysin. Linker amino acids between EADs and CBDs were generated through the ab initio method, resulting in complete lytic protein with both EAD and CBDs. Group VI lysin exhibited structural fluctuation but stabilized after 45 ns (Fig. 3G and H). Sequence alignment and structural superimposition of the 3D structures of both EADs revealed fully conserved catalytic residues (D10, D92, E94, D182) (Fig. 8).

#### 3.3.5 Other group lysins

For Group IV lysins, homology modeling was not possible due to the absence of a suitable template or crystal structure. However, a 3D structure for the representative Group IV endolysin (ADT64066.1) was later generated using AlphaFold3. Sequence alignment and structural superimposition of this model with PDB 4IWT, the cell wall-binding domain of the LytA protein from *S. pneumoniae*, revealed a high degree of similarity in the CBD domain (Supplementary Figure S1). Similarly, no templates were available for the Prophage_tail domains of Group VII and VIII lysins, so their 3D structures were also generated using AlphaFold3 (Supplementary Figures S2 and S3). Due to the lack of appropriate templates, superimposition analysis was not performed for these lysins.

## 4 Discussion

*S. pneumoniae* is the fourth most fatal pathogen and a leading contributor to an estimated 450 million pneumonia cases every year globally [50,51]. *S. pneumoniae* causes 27.3 % of community-acquired pneumonia [51]. The misuse and overuse of antibiotics have led to the emergence of multidrug-resistant strains, such as 23 F, 19 A, 19 F, and 14, which are associated with severe infections [52,53]. In the United States, pneumococcal resistance rates range from 13.8 to 41.8 % for penicillin, 20–40 % for macrolides, 25.9 % for tetracyclines, 25–45 % for trimethoprim-sulfamethoxazole, 21.8 % for lincosamides, and 1–2% for fluoroquinolones [21]. The growing threat of pneumococcal antibiotic resistance has driven the search for alternative treatments, including bacteriophage-derived endolysins.

**Fig. 9:**
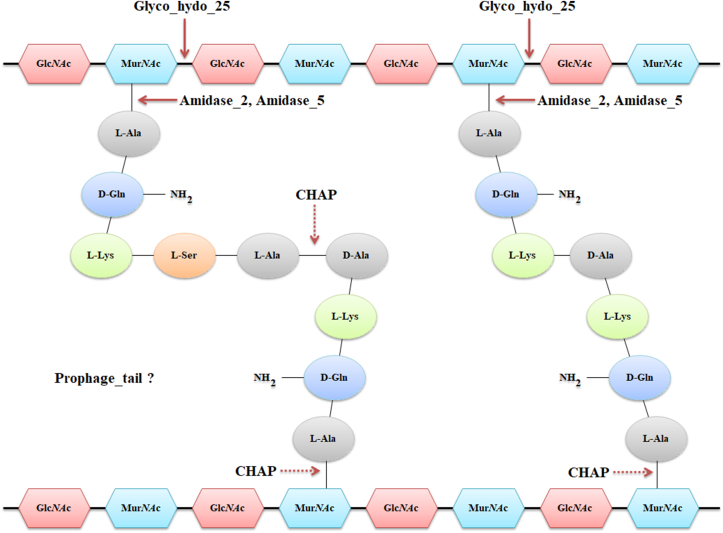
Catalytic sites of *S. pneumoniae* phage endolysins throughout the pneumococcal cell wall. Solid arrows indicate proven cleavage sites whereas dashed arrows indicate possible cleavage sites. The function of Prophage_tail is still unknown.

*Streptococcus* phages of the *Siphoviridae* family are predominant in nature. Each *S. pneumoniae* phage typically contains one endolysin, though eight phages have been identified with an additional endolysin. Eight groups were sorted through phylogenetic analysis based on their enzymatically active domain. The primary EADs include CHAP, Amidase_5, Glyco_hydro_25, Prophage_tail as EAD, while their CBDs include CW_binding_1 and CW_7. The lysin member of these groups contains a single EAD, but the number of CBDs varied from group to group. The six CW_binding_1 repeats are conserved in Groups I, II, and IV lysins. In contrast, Group V’s CW_binding_1 domain is notably different, with only a small section aligning with LytA. While Groups I, II, and IV demonstrate high conservation throughout the LytA CBD, Group V stands out as an exception despite having a similar CBD domain. The largest group, Group I, is characterized by endolysins containing an Amidase_2 domain and six repeats of the CW_binding_1. These proteins show high sequence similarity to the autolysin LytA, a protein in *S. pneumoniae* responsible for cell wall degradation under specific conditions, such as nutrient depletion or antibiotic treatment rather than during normal growth [54]. The Amidase_2 domain functions as an N-acetylmuramoyl-l-alanine amidase, cleaving the bond between NAM and the first l-alanine of the peptide stem, similar to the action of Amidase_5 in Group IV (Fig. 9) [55]. Despite their similar function, these domains differ structurally, with Amidase_2 has a unique fold and a conserved catalytic triad, while Amidase_5 exhibits distinct variations in secondary elements and substrate-binding residues. Some well characterized *Streptococcal* endolysins, such as Pal, hbl, Ejl, fall under Group IV. Groups V and VI contain endolysins with a Glyco_hydro_25 domain, a lysozyme that hydrolyzes 1,4-β-linkages between N-acetylglucosamine (NAG) and N-acetylmuramic acid (NAM) in peptidoglycan layer. Group III endolysins possess a CHAP domain, a peptidase that may cleave either cross-bridged amino acids or the bond between NAM and l-alanine in the stem peptide [55]. Though the CHAP domain in *S. pneumoniae* has not been fully characterized, it is believed to function similarly to the CHAP domain in the PcsB protein [56]. The Prophage_tail EAD, found in some endolysins, remains poorly understood, and its cleavage site in the peptidoglycan layer has yet to be identified (Fig. 9).

Molecular dynamics simulation of the three-dimensional structures of Group I, II, III and VI endolysins have provided insights into their structural stability and functional characteristics when compared to superimposed template structures (Fig. 3). Group I endolysins, containing the Amidase_2 domain, showed 100 % sequence similarity with LytA of the host bacterium. Both proteins are Zn^2+^ dependent, and all catalytic and Zn^2+^ binding residues are fully conserved in Group I lysin and LytA protein. Structural superimposition of EAD of Group II lysin with PlyPy and LysK proteins showed conservation of the active site and Ca^2+^ binding residues. Sequence alignment and superimposition of the CBD with LytA’s CW_binding_1 module showed the possibility of finding a new repeat in Group II lysins (Fig. 6D). While CBD of LytA contains six CW_binding_1 repeats, with aromatic residues in these repeats forming specific bonds with choline. Notably, two tryptophan residues (W), aromatic in nature, form the first repeat form a pi-cation interaction with a choline molecule present between the 1st and 2nd repeats. Another aromatic residue, tyrosine (Y), located in the 2nd repeat, sometimes forms an additional pi-cation interaction with the choline [57]. Interactions of choline with the last two repeats of LytA, and the 5th repeat and the probable 6th repeat of Group II endolysin were observed (Fig. 6A and B). Different types of interactions were formed between the tryptophan residues in the 5th repeat and the tyrosine residue of the following β strand of Group II endolysin. The same amino acids also participate in the interaction between the 5th and 6th repeat of CBD in LytA. Molecular docking further supports the presence of the 6th CBD repeat in Group II endolysin, suggesting its presence and functional importance. The CBD repeats play important roles in both the structural stabilization and endolysin activity [58]. Deletion of CBD repeats significantly diminishes ligand recognition and, consequently, endolysin activity. Additionally in *S. pneumoniae,* an increase in the number of cell wall binding repeats enhances the choline-binding capacity of endolysin, which ultimately increases endolysin activity [57]. Therefore, the newly identified 6th repeat in the CBD of Group II lysin may contribute to its functionality by reinforcing both structural stability and activity. Group III lysins have 99.16 % sequence similarity with LysME-EF1, a protein from *E. faecalis*, containing a CHAP domain and a CBD domain. Until now, no computational database has identified the presence of a CBD domain in Group III lysins. LysME-EF1 is a multimeric endolysin that consists of one full-length protein and three additional N-terminally truncated CBD peptides. These additional peptides facilitate the lytic activity of LysME-EF1 and have been to work against several *E. faecalis* strains [49]. The high sequence similarity between Group III lysins and LysME-EF1 raises several important questions which need to be addressed in future experiments: a) Did Group III lysins and LysME-EF1 co-evolve? b) Is Group III lysin a multimeric endolysin similar to LysME-EF1? c) Can Group III lysins act with additional CBDs when used as antimicrobials against pneumococci? d) Does Group III lysin possess the capability to act as a broad-spectrum antimicrobial, hydrolyzing both pneumococci and *E. faecalis*? Group VI lysins contain a unique CBD module comprising three tandem repeats (CW_7) [59]. Each repeat, consisting of 42 amino acids, functions as a cell wall binding module, facilitating the degradation of choline or ethanolamine in the cell wall [60]. The CW_7 domain binds to the *NAG*-*NAM*-L-Ala-D-isoGln chain, indicating that both the glycan chain and the stem peptide are essential for stabilizing CBD/ligand interactions [61]. These three tandem repeats act cooperatively; however, the deletion of one or two repeats does not eliminate the formation of a structurally active cell wall binding domain [61]. Modular recombination of proteins plays an important role in the evolution of cell wall hydrolyzing enzymes, enabling rapid adaptation [62,63]. This phenomenon has been experimentally confirmed by constructing chimeric endolysins, which retain full functionality in hydrolyzing bacterial cell walls [64,65]. Our study sheds light on the diversity of modular domains present in *S. pneumoniae* endolysins and their potential for future therapeutic applications.

## 5 Conclusion

The rapid and unchecked emergence of antibiotic resistance of bacterial pathogens poses a major threat to human health around the world. The emergence of the resistant gene in these bacterial pathogens is leading from multidrug resistance to superbug formation. Some *S. pneumoniae* are multidrug-resistant that can lead to treatment failures. Bacteriophage-derived endolysins can play a crucial role to be used as an alternative or adjunct to antibiotics. We have exploited *S. pneumoniae* phage genomes to generate an endolysin database for molecular modelling and diversity analysis of these lytic proteins. Phylogenetic analysis showed eight distinct endolysin groups and each group have a different domain architecture. Sequence analysis showed conserved catalytic and metal-binding residues. An additional cell wall binding repeat in Group II lysin was also observed, which was not described previously. The comparative computational analysis identified the existence of CBD in Group III lysin. The newly identified repeat and CBD of Group II and Group III lysin, respectively, may play a crucial role in their activity. The current study provides the first insight into the molecular and diversity analysis of *S. pneumoniae* phage endolysins that could be valuable for developing novel lysin-based therapeutics. However, further *in vitro* and *in vivo* studies are essential to experimentally validate these findings and unravel their roles as potential treatments for MDR bacterial infections.

## CRediT authorship contribution statement

**Tahsin Khan:** Writing – original draft, Methodology, Investigation, Formal analysis, Conceptualization. **Shakhinur Islam Mondal:** Writing – review & editing, Writing – original draft, Visualization, Supervision, Resources, Project administration, Methodology, Investigation, Funding acquisition, Formal analysis, Conceptualization. **Araf Mahmud:** Methodology, Investigation, Formal analysis. **Daniyal Karim:** Formal analysis, Data curation. **Lorraine A. Draper:** Writing – review & editing. **Colin Hill:** Writing – review & editing. **Abul Kalam Azad:** Writing – review & editing, Investigation. **Arzuba Akter:** Writing – review & editing, Writing – original draft, Supervision, Resources, Project administration, Methodology, Investigation, Formal analysis, Conceptualization.

## Ethical approval

Not required.

## Funding

Research Center of Shahjalal University of Science and Technology, Grant/Award Number: LS/2021/1/17.

## Declaration of competing interest

The authors declare that they have no known competing financial interests or personal relationships that could have appeared to influence the work reported in this paper.

## Figures and Tables

**figs1:**
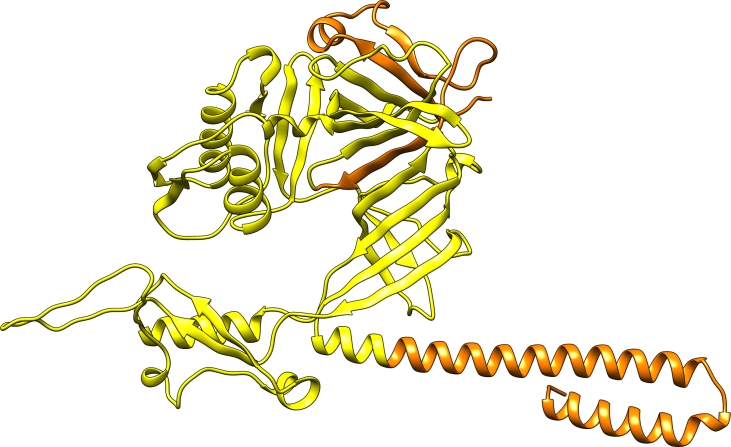
**Supplementary figure S2:** 3D structure of Group VII lysin. The yellow color indicates the Prophage_tail region (59-396) within the lysin.

**figs2:**
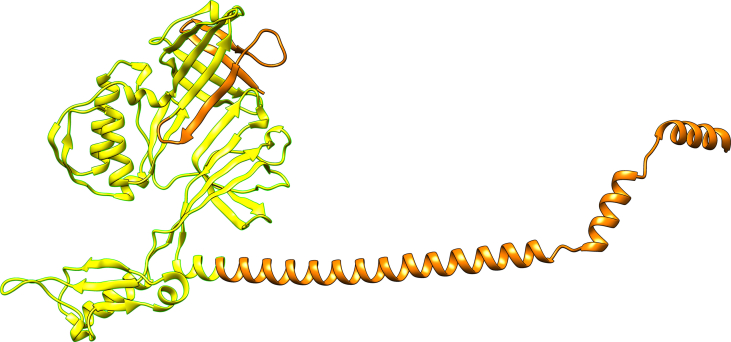
**Supplementary figure S3:** 3D structure of Group VIII lysin. The yellow color indicates the Prophage_tail region (38-366) within the lysin.

## Appendix A Supplementary data

Multimedia component 1 Multimedia component 1

Multimedia component 2 **Supplementary figure S1:** Superimposition and sequence similarity between LytA CBD domain (PDB 4IWT) and Group IV lysin. (A) Blue and orange colour indicates Group IV lysin and LytA CBD domain, respectively. (B) Alignment of CBD of LytA of *S. pneumoniae* and Group IV lysin (ADT64066.1). Multimedia component 2
