# Supplementary figures and images for: Identification of cell wall binding domains and repeats in Streptococcus pneumoniae phage endolysins: A molecular and diversity analysis
Source: Biochem Biophys Rep. 2024 Oct 14;40:101844. doi: 10.1016/j.bbrep.2024.101844 (PMC11525621; doi:10.1016/j.bbrep.2024.101844)

(A)

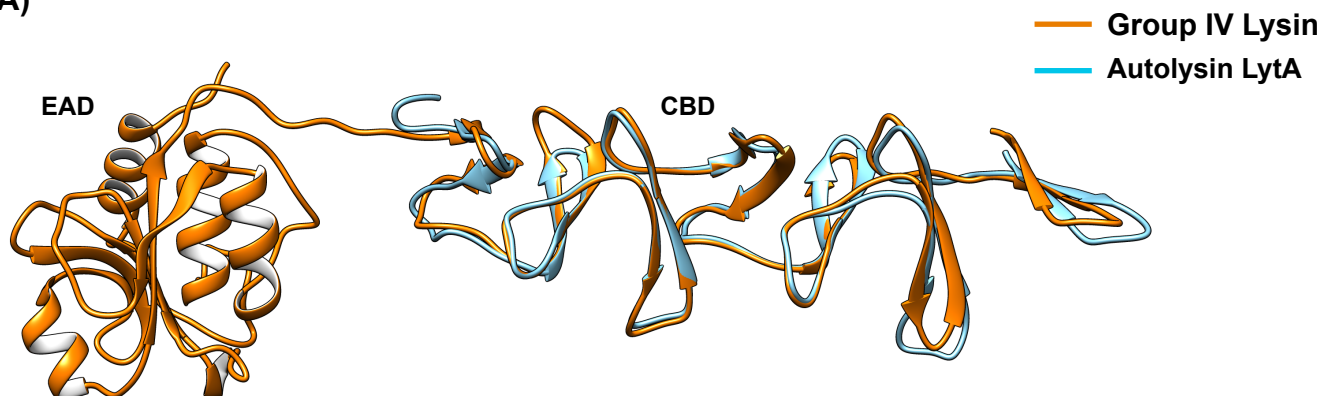

(B)

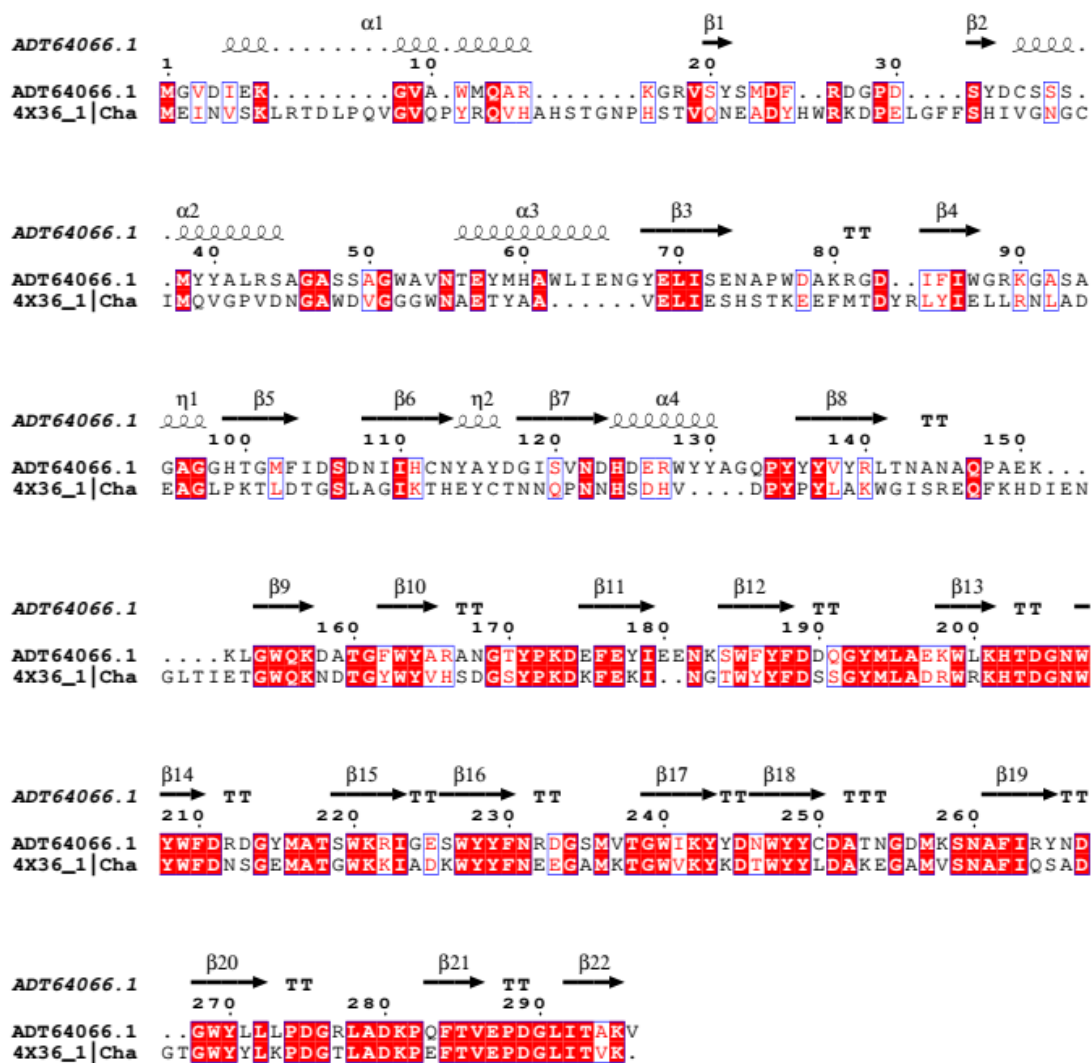

Supplement: Multimedia component 2 — Supplementary figure S1: Superimposition and sequence similarity between LytA CBD domain (PDB 4IWT) and Group IV lysin. (A) Blue and orange colour indicates Group IV lysin and LytA CBD domain, respectively. (B) Alignment of CBD of LytA of S. pneumoniae and Group IV lysin (ADT64066.1). [file mmc2.pdf]
